# Anti-Obesity Effect of *Lactobacillus acidophilus* DS0079 (YBS1) by Inhibition of Adipocyte Differentiation through Regulation of p38 MAPK/PPARγ Signaling

**DOI:** 10.4014/jmb.2402.02012

**Published:** 2024-03-29

**Authors:** Youri Lee, Navid Iqbal, Mi-Hwa Lee, Doo-Sang Park, Yong-Sik Kim

**Affiliations:** 1 Department of Microbiology, College of Medicine, Soonchunhyang University, Cheonan 31151, Republic of Korea; 2 Institute of Tissue Regeneration, College of Medicine, Soonchunhyang University, Cheonan 31151, Republic of Korea; 3 Nakdonggang National Institute of Biological Resources, Sangju 37242, Republic of Korea; 4 Biological Resource Center, Korea Research Institute of Bioscience and Biotechnology, Jeongeup 56212, Republic of Korea

**Keywords:** *Lactobacillus acidophilus* YBS1, anti-adipogenesis, PPARγ, p38 MAPK, obesity

## Abstract

Obesity is spawned by an inequality between the portion of energy consumed and the quantity of energy expended. Disease entities such as cardiovascular disease, arteriosclerosis, hypertension, and cancer, which are correlated with obesity, influence society and the economy. Suppression of adipogenesis, the process of white adipocyte generation, remains a promising approach for treating obesity. Oil Red O staining was used to differentiate 3T3-L1 cells for screening 20 distinct *Lactobacillus* species. Among these, *Lactobacillus acidophilus* DS0079, referred to as YBS1, was selected for further study. YBS1 therapy decreased 3T3-L1 cell development. Triglyceride accumulation and mRNA expression of the primary adipogenic marker, peroxisome proliferator-activated receptor gamma (PPARγ), including its downstream target genes, adipocyte fatty acid binding protein 4 and adiponectin, were almost eliminated. YBS1 inhibited adipocyte differentiation at the early stage (days 0–2), but no significant difference was noted between the mid-stage (days 2–4) and late-stage (days 4–6) development. YBS1 stimulated the activation of p38 mitogen-activated protein kinase (p38 MAPK) during the early stages of adipogenesis; however, this effect was eliminated by the SB203580 inhibitor. The data showed that YBS1 administration inhibited the initial development of adipocytes via stimulation of the p38 MAPK signaling pathway, which in turn controlled PPARγ expression. In summary, YBS1 has potential efficacy as an anti-obesity supplement and requires further exploration.

## Introduction

Obesity, which is restricted as having a body mass index of 30 kg/m^2^ or more, is a significant public health interest. It is described by excess fat accretion in the adipose tissue of the body owing to an imbalance between energy increment and expenditure. Obesity is a complicated disease substance swayed by physiological, environmental, and genetic factors, including habits such as consuming high-calorie meals and living an immobile activity [1-4]. Obesity is associated with numerous serious health outcomes such as high blood pressure, diabetes mellitus type 2, non-alcoholic fatty liver disease, illnesses affecting the muscles and bones, heart conditions, and certain forms of cancer. Obesity is a prevalent problem that affects people of all age groups. The global population of overweight or obese children and youth aged 5 to 19 years was 330 million in 2016, with an additional 40 million children under the age of 5 years also being affected by this affliction [5-8]. By 2025, the global obesity rate is expected to reach more than 20% among adults.

To date, studies on adipocyte biology have been a primary focus in the fight against obesity. Adipogenesis refers to the strictly planned and regulated process of adipocyte differentiation, which involves the transformation of premature adipocytes into mature adipocytes. Adipogenesis involves several important regulators. Adipocyte differentiation involves multiple stages involving alterations in hormone responsiveness and morphology. This process is regulated by transcription factors and signaling networks. Identifying regulatory chemicals and processes that limit adipocyte differentiation or proliferation may be crucial to preventing obesity [9, 10]. The process of adipogenesis is complex and involves the transformation of pre-adipocytes into adipocytes, which can store lipids [11, 12]. Hormonal stimulation is necessary at this stage. Adipogenesis can be categorized into three stages: early, middle, and late [13, 14]. During adipocyte development initiation, there is an upregulation in CCAAT/enhancer-binding protein α (C/EBPα) expression, which eventually leads to the expression of peroxisome proliferator-activated receptor (PPARγ) and CCAAT/enhancer-binding protein δ (C/EBPδ). Pre-adipocytes have the ability to differentiate into fully developed adipocytes, which are characterized by the existence of lipid droplets [15, 16]. The mitogen-activated protein kinase (MAPK) signaling pathway, which includes ERK, p38, and c-Jun N-terminal kinase, is essential for multiple cellular activities, including adipocyte formation [17-19]. Currently, multiple medications are available for the treatment of obesity and its affiliated health issues. Although they possess potential advantages, they can also induce adverse effects, including nausea, sleeplessness, gastrointestinal issues, stomach ailments, and cardiovascular concerns. The discovery of novel target molecules and medicines that can efficiently control adipocyte differentiation has the plausible to be advantageous for the development of innovative therapies for corpulence and other metabolic disorders.

Recently, the microbiome has been highlighted in diverse fields such as food, pharmaceutical, and even medical industries. The gut microbiome forms of varied microorganisms including bacteria, viruses, bacteriophages, and archaea and it needs to be strictly balanced and maintained for beneficial outcomes to their host. Probiotics, living organisms that produce metabolites from dietary compounds and have beneficial effects, have been extensively studied and are widely utilized as functional food ingredients owing to their diverse health-enhancing properties. The new term, pharmabiotics, has been coined because certain microbiomes can be used as therapeutic drugs in special disease [20]. The gut microbiome is acknowledged as a significant contributor to the progression of metabolic illnesses such as obesity. Our previous study revealed that the beneficial microbiome, *Bifidobacterium* from infant feces, ameliorates high-fat diet (HFD)-induced obesity in animal models via promoting browning and inhibiting adipogenesis [21]. Therefore, beneficial microbes are strong candidates as alternative pharmaceutical drugs for treating obesity.

*Lactobacillus* species are commonly used as dietary supplements and functional meals, purportedly enhancing metabolic activity and protecting against obesity and its associated ailments. In recent years, the effects of lactic acid bacteria (LAB) on obesity and related metabolic disorders have received considerable attention. *Lactobacillus rhamnosus* has various medical applications, including anti-obesity properties and the ability to combat resistant bacteria such as vancomycin-resistant enterococci [22]. *Lactobacillus reuteri* can organically inhabit the digestive system, particularly after dairy product consumption. Infants can potentially acquire this strain of *Lactobacillus* through breastfeeding [23, 24]. *Lactobacillus acidophilus* is a common ingredient in yogurt, health foods, and medications. *L. acidophilus* is beneficial for lowering cholesterol, treating and preventing diarrhea, controlling the immune system, and slowing the spread of cancer and other health-related issues [25]. Furthermore, two LAB strains, *Bifidobacterium longum* DS0956, and *L. rhamnosus* DS0508, stimulated browning and lipolysis both in vitro and in vivo [26]. The present study aimed to explore the effect of YBS1 on obesity through the inhibition of adipocyte development. Our findings suggested that YBS1 has the possible to be used as a medicinal factor against obesity.

## Materials and Methods

### Bacterial Culture and Preparation of Bacterial Cell-Free Supernatants

The LAB strains were obtained from the Bio R&D Product program (https://biorp.kribb.re.kr/) and Korean Collection for Type Cultures (Republic of Korea). The bacterial strains were cultivated in de Man, Rogosa and Sharpe media (BD, USA) under anaerobic conditions at 37°C for 36 h. The bacterial cultures were incubated at 65°C for 30 min for pasteurization and centrifuged at 3,000 ×*g* for 10 min. Each of the cell-free supernatants was collected in a fresh new tube and stored at -70°C until use. The same cell-free supernatants were freeze-dried and then dissolved in 10% volume of distilled water for 10× samples. A list of all the LAB used in this study is offered in Table S1.

### Chemicals, Antibodies, and Kits

High-glucose Dulbecco’s modified Eagle’s medium (DMEM) and fetal bovine serum were purchased from Atlas Biologicals (Fort Collins, USA). Dexamethasone, insulin, isobutylmethylxanthine, 3-(4,5-dimethylthiazol-2-yl)-2,5-diphenyltetrazolium bromide (MTT), dimethyl sulfoxide (DMSO), rosiglitazone, Oil Red O (ORO) powder, 4% formaldehyde, and SB203580 were acquired from Sigma-Aldrich (USA). Antibodies against adiponectin, adipocyte fatty acid binding protein 2 (aP2), p38 MAPK, p-p38 MAPK, anti-mouse IgG antibody conjugated with horseradish peroxidase, and anti-rabbit IgG antibody conjugated horseradish peroxide were purchased from Cell Signaling Technology (USA). Antibodies against PPARγ and β-actin were obtained from Santa Cruz Biotechnology (USA). A bicinchoninic acid protein assay kit was purchased from Thermo Fisher Scientific (USA), and western enhanced chemiluminescence substrate and protein-loading buffer were purchased from Bio-Rad Laboratories, Inc. (USA).

### Cell Culture, Differentiation, and Treatment

The 3T3-L1 pre-adipocytes (ATCC CL-173) were purchased from the American Type Culture Collection (USA) and cultured at 37°C in a 5% CO_2_ incubator. Prior to differentiation, cells were cultured in DMEM containing 10% heat-incubated newborn calf serum (Atlas Biologicals) and 1% penicillin-streptomycin. During differentiation, the cells were cultured in DMEM containing 10% heat-incubated fetal bovine serum and 1%penicillin-streptomycin. The 3T3-L1 cells were cultured through treatment with 0.5 mM 3-isobutyl-1-methylxanthine, 1 mM dexamethasone, and 10 μg/ml insulin (MDI) in DMEM supplemented with 10% fetal bovine serum. The cells were harvested after 6 d, and the medium was changed every 2–3 d. YBS1 and SB203580 (a p38 MAPK inhibitor) were co-administered according to the target timeline to confirm chemical inhibition.

### Cell Viability Assay

After achieving 70–80% confluence, 3T3-L1 pre-adipocytes were placed in 24-well plates at a density of 1 × 10^4^ cells per well. Consequently, differentiation was initiated, and cells were subjected to treatment with YBS1 at a concentration of 5 μl/ml for 24 and 48 h, as well as for 1, 3, and 5 d. The control group was not subjected to culture cocktails, and the medium was replaced every 2 d. A 50 μl MTT solution with a concentration of 5 mg/ml was introduced and maintained at a temperature of 37°C for a 4 h duration. The formazan crystals were dissolved in 200 μl DMSO, and the absorbance was quantified at 590 nm using a Victor X3 multilabel reader (Perkin Elmer, USA).

### ORO Staining

The 3T3-L1 pre-adipocytes were cultured in 6-well plates and led to differentiate for 6 d to stain triglycerides (TGs) and lipids. To preserve their structures, differentiated cells were rinsed twice with 1× phosphate-buffered saline and treated with 10% formalin for 1 h. The cells were treated with a purified 0.3% ORO solution for 15–30 min. The stained cells were rinsed four times with distilled water, and any visible changes in their characteristics were captured using an Axiovert-25 microscope (Carl Zeiss, Germany). The cells that were stained for ORO quantification were washed with 100% isopropanol, and the absorbance was measured at 520 nm using a Victor X3 multilabel reader (Perkin Elmer).

### Quantitative Reverse Transcription Polymerase Chain Reaction (qRT-PCR)

The total RNA was obtained by harvesting 3T3-L1 pre-adipocytes after differentiation, and RNA was extracted using an RNA extraction kit (Qiagen, USA). Using a Maxime RT PreMix Kit (Intron Biotechnology, Republic of Korea) on a Veriti 96-Well Thermal Cycler (Applied Biosystems, Singapore), 1 μg of RNA was used for cDNA synthesis via qRT-PCR. The qRT-PCR analysis was conducted using the CFX96 RT-PCR detection equipment (Bio-Rad, Singapore) and the iQ SYBR Green Supermix Kit (Bio-Rad). The collected data were standardized using TATA binding protein as the reference. The primer sequences used in this study are listed in Table S2.

### Western Blot Analysis

The 3T3-L1 pre-adipocytes were rinsed twice with chilled 1× phosphate-buffered saline and then lysed using RIPA buffer containing a protease inhibitor cocktail, phenylmethylsulphonyl fluoride, and sodium orthovanadate (USA). The proteins were obtained through gentle separation and then stored at a temperature of 4°C for a 15 min duration. Subsequently, the mixture was centrifuged at 4°C and 12,000 ×*g* for 15 min. Only the supernatants were retained. Total protein was quantified using the Bicinchoninic acid Assay Kit (Thermo Fisher Scientific). The experiment involved loading equal quantities of protein onto a 4–20% sodium dodecyl sulfate-polyacrylamide gradient gel (Mini-PROTEAN Precast Gel, Bio-Rad) and subsequently transferring to a polyvinylidene difluoride membrane (Trans-Blot SD Semi-Dry Cell, Bio-Rad) using semi-dry power (Bio-Rad) at 15 V for a duration of 1 h. The membranes were blocked at 25°C for 1 h using a 1% solution of bovine serum albumin or skim milk in 1× Tris-buffered saline containing 0.1% Tween-20 (TBST). Subsequently, a primary antibody was applied and left overnight at 4°C. The membranes underwent three rounds of washing, each lasting for 5 min, using a TBST solution. Subsequently, a secondary antibody was added and incubated at room temperature for 1 h. Finally, the membranes were washed three times, each for 5 min, with TBST solution. The proteins were identified using the enhanced chemiluminescence substrate (Bio-Rad) and quantified using ChemiDoc with ImageLab 2.0 (Bio-Rad). The data values were quantified using Image J software, with the anti-β-actin antibody serving as a loading reference. PPARγ was diluted at a ratio of 1:500, whereas adiponectin, aP2, C/EBPα, p38 MAPK, p38 MAPK, and β-actin were diluted at a ratio of 1:1000. Secondary antibodies coupled with horseradish peroxidase were diluted at a ratio of 1:3000. Primary and secondary antibodies were diluted in a solution containing 1% bovine serum albumin or skim milk.

### Statistical Analysis

Data are presented as the mean ± standard error. To analyze the results, a one- or two-way analysis of variance was conducted, followed by the post hoc Bonferroni test using GraphPad Prism 8 software (GraphPad Software, USA). Differences were considered significant at * *p* < 0.05, ** *p* < 0.01, and *** *p* < 0.001.

## Results

### YBS1 Reduces TG Accumulation in 3T3-L1 Pre-Adipocytes Differentiation

**Fig. 1:**
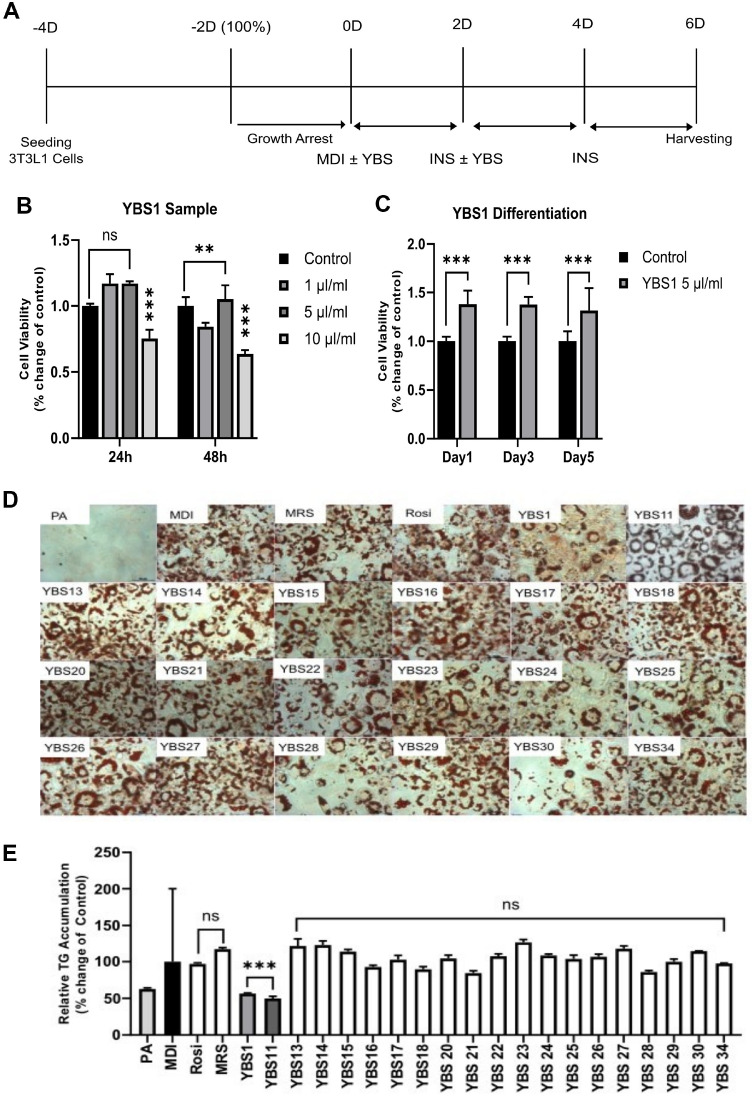
Cell viability and triglyceride (TG) accumulation in 3T3-L1 pre-adipocytes with YBS1 cell-free supernatant treatment. (**A**) Schematic diagram of 3T3-L1 pre-adipocyte differentiation with YBS1 treatment. (**B**) Cell viability was evaluated using an MTT assay 24 and 48 h after treatment with YBS1 (1, 5, and 10 μl/ml). (**C**) Cell viability was measured in 3T3-L1 pre-adipocyte during differentiation using an MTT assay 1, 3, and 5 days after treatment with 5 μl/ml YBS1. (**D**) ORO staining for TG accumulation in differentiated 3T3-L1 pre-dipocytes with 20 LAB strains at day 6. (**E**) Quantification of TG accumulation shown in (D). Data from a minimum of three experiments are presented as average ± standard error of mean. ns, non-significant; *p < 0.05, ** *p* < 0.01, *** *p* < 0.001; MDI (control) vs. treatment groups. MDI: 0.5 mM isobutylmethylxanthine, 1 μM dexamethasone, and 10 μg/ml insulin.

We used the cell-free supernatant of 20 *Lactobacillus* strains isolated from newborn feces and ORO staining to determine the effect of LAB on obesity (Fig. S1). Based on the most effective inhibition of TG accumulation by LAB treatment, we selected YBS1 for further investigation. The work flowchart followed for adipocyte differentiation is depicted in Fig. 1A. An MTT assay was conducted 24 and 48 h after treatment with 1, 5, and 10 μl/ml of cell-free supernatant to evaluate cell viability with the YBS1 treatment (Fig. 1B). Treatment with YBS1 cell-free supernatant at 1 and 5 μl/ml did not cause any cytotoxicity. However, after 24 and 48 h, treatment with 10 μl/ml YBS1 showed notable toxicity in contrast to the untreated control. Therefore, 5 μl/ml of YBS1 was selected for the subsequent tests. We measured cytotoxicity on days 1, 3, and 5 after treating with 5 μl/ml YBS1 in accordance with the differentiation time points (Fig. 1C). We did not observe any harmful effects of the YBS1 treatment (Fig. 1D). Consequently, in this investigation, the maximum YBS1 concentration was established as 5 μl/ml, and subsequent research was conducted at this concentration. In summary, 100% 3T3-L1 pre-adipocytes were obtained following seeding and processing with MDI cocktails and were harvested in accordance with the intended use.

To induce adipocyte differentiation, 5 μl/ml of YBS1 was administered to 3T3-L1 pre-adipocytes handled with MDI, with rosiglitazone and de Man, Rogosa and Sharpe medium serving as the positive and negative controls, respectively. Based on the results of ORO staining, YBS1 and YBS11 (*Lactobacillus paracasei*) had a significant effect in lowering TG buildup. Therefore, we further examined YBS1 in the context of anti-obesity effects (Fig. 1D and E). The well-known probiotic *Lactobacillus rhamnosus* GG (LGG) is used as a dietary supplement for several conditions, including obesity. Therefore, we compared the effect of YBS1 to that of LGG on the suppression of TG accumulation in 3T3-L1 pre-adipocytes. The data presented in Fig. S2A and S2B show that YBS1 inhibited TG accumulation more significantly than LGG and the effect of LGG was negligible.

### YBS1 Treatments Alter the Expression of Adipogenic- and Lypolytic-Specific Markers during Adipogenesis

**Fig. 2:**
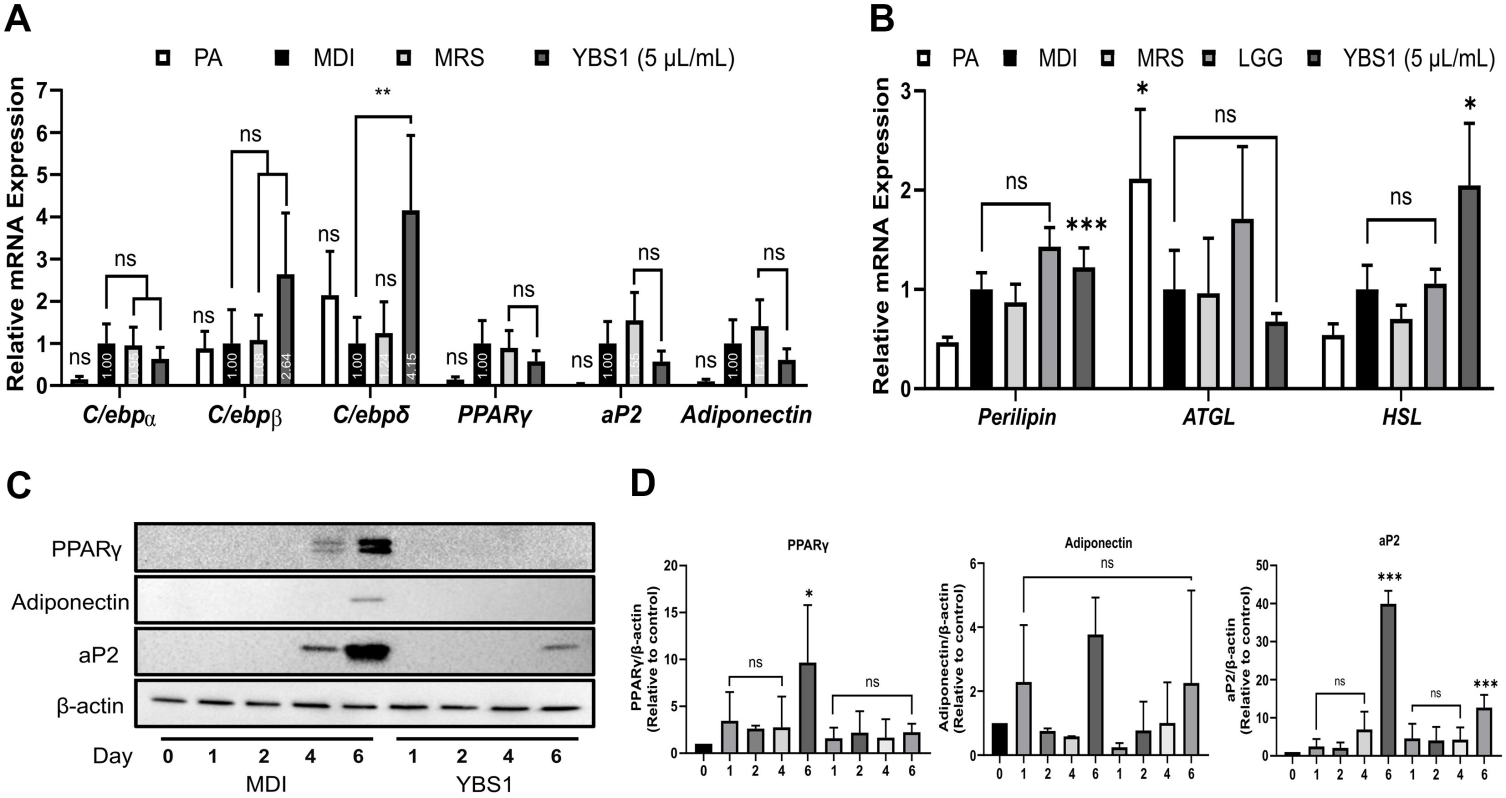
Alterations in the adipogenic and lipolytic-related gene expression levels in differentiated 3T3-L1 pre-adipocytes with YBS1 cell-free supernatant treatment. (**A**) The mRNA expression levels of adipogenic markers (C/ebpα, C/ebpβ, C/ebpδ, Pparγ, aP2, and adiponectin) with YBS1 treatment measured via qRT-PCR were compared to those with MDI treatment. (**B**) The mRNA expression levels of lipolysis markers (perilipin, ATGL, and HSL). (**C**) Protein expression levels of adipogenic markers (aP2, adiponectin, and PPARγ) were measured using western blot. TATA binding protein and β- actin were used as the mRNA and protein loading controls, respectively. (**D**) Quantification of the protein expression in (**C**). Data were significant at *p* < 0.05, MDI (control) vs. treatment groups. MDI: 0.5 mM isobutylmethylxanthine, 1 μM dexamethasone, and 10 μg/ml insulin.

We tested the expression of adipogenesis-specific markers in 3T3-L1 pre-adipocytes to elucidate the mechanism underlying lipid accumulation suppression during adipogenesis through treatment with YBS1. The mRNA expression of late adipogenic markers aP2 and adiponectin, as well as of C/EBPα, C/EBPβ, C/EBPδ, and PPARγ, was evaluated, as illustrated in Fig. 2A. The mRNA expression of C/EBPβ (2.64-fold) and C/EBPδ (4.15-fold) was marginally increased in YBS1-treated 3T3-L1 pre-adipocytes. Moreover, the YBS1 treatments decreased the mRNA expression of important adipogenic symbols such as PPARγ (0.58-fold) and its downstream target genes aP2 (0.57-fold) and adiponectin (0.61-fold) (Fig. 2A). Next, the expression of genes linked to lipolysis, including hormone-sensitive lipase (HSL), adipose TG lipase (ATGL), and perilipin, was explored. Perilipin (1.22-fold) and HSL (2.05-fold) mRNA expression levels were elevated following YBS1 treatment (Fig. 2B). However, as presented in Fig. 2B, a discernible change in ATGL mRNA expression status was absent. Furthermore, to determine the precise time point at which YBS1 treatment influenced the differentiation of adipocytes, we investigated the protein expression levels at 0, 1, 2, 4, and 6 d (Fig. 2C). On day 4, MDI treatment markedly increased the expression of PPARγ and downstream gene, aP2, which peaked on day 6. Furthermore, on day 6 of MDI treatment, the terminal adipogenic marker adiponectin was detected. However, on days 4 and 6 of the YBS1 treatment, a considerable decrease in the expression of PPARγ and aP2 was noted. Additionally, we observed that in 3T3-L1 pre-adipocytes treated with YBS1, adiponectin expression was completely eliminated on day 6 (Fig. 2C and D). Thus, YBS1 administration during 3T3-L1 differentiation decreases PPARγ expression, a critical adipogenic marker, and its downstream genes, including adiponectin.

### YBS1 Treatment Reduces TG Accumulation in the Early Stages of 3T3-L1 Pre-Adipocyte Differentiation

**Fig. 3:**
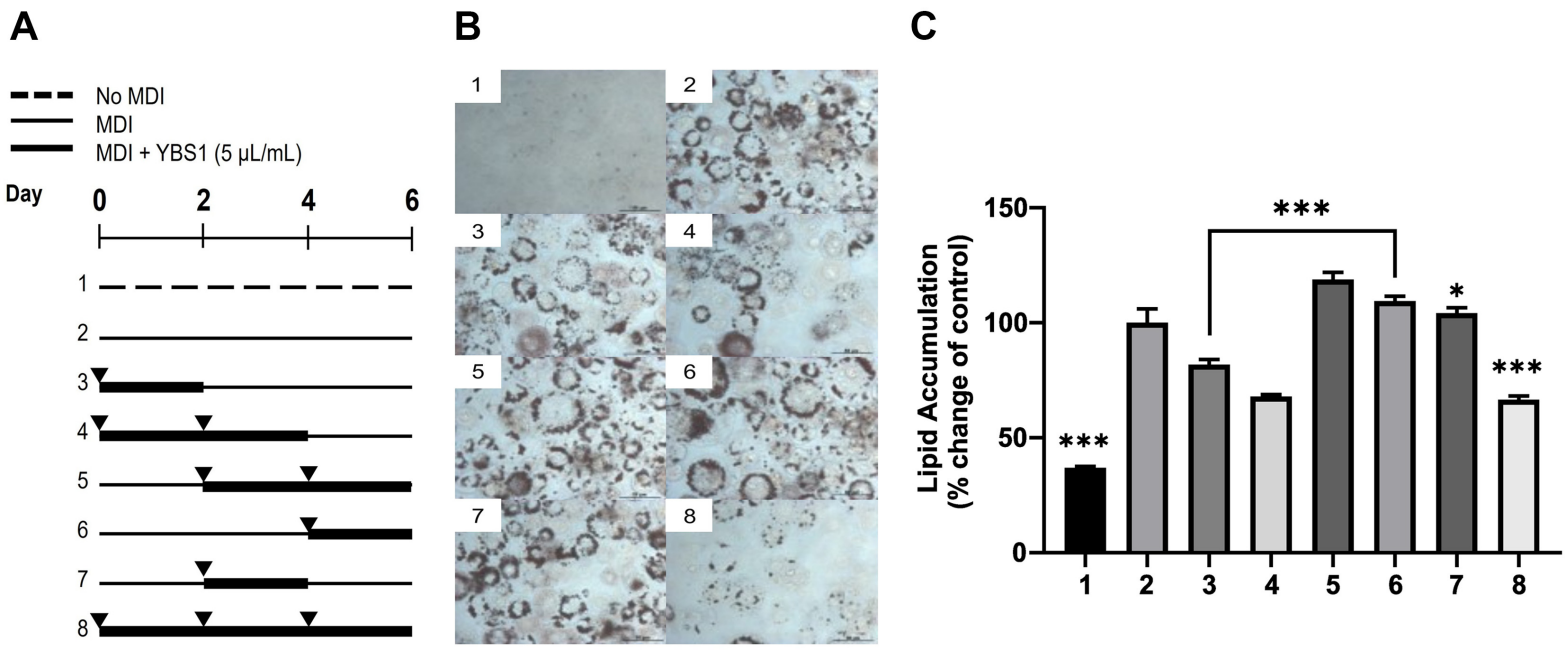
Inhibition effect on early adipogenesis through YBS1 treatment (5 μl/ml) in 3T3-L1 pre-adipocytes. (**A**) Schematic diagram of the effect of YBS1 treatment during adipogenesis at the specified time points. (**B**) Photographs of cells taken at 40× magnification of ORO staining at the specified adipocyte differentiation time points. (**C**) Quantification of ORO staining. MDI: 0.5 mM isobutylmethylxanthine, 1 μM dexamethasone, and 10 μg/ml insulin. ns, *, **, and *** indicate non-significance, *p* < 0.05, *p* < 0.01, and *p* < 0.001, respectively.

The development of 3T3-L1 pre-adipocytes or adipogenesis occurs in three separate stages: early, intermediate, and late. Dexamethasone, isobutylmethylxanthine, and insulin are used to induce the early stages of adipogenesis. Subsequently, mitotic clonal expansion (MCE), a synchronized process in growth-arrested 3T3-L1 pre-adipocytes, occurs. The treatment groups were divided into three stages according to the adipocyte differentiation processes, early (0–2 days), middle (2–4 days), and late (4–6 days), to determine the time point in 3T3-L1 pre-adipocyte development that is influenced by treatment with YBS1 5 μl/ml (Fig. 3). The data presented in Fig. 3A and 3B suggest that a considerable reduction in TG accumulation was present in the YBS1 treatment groups (nos. 3, 4, and 8) during the early phases of adipogenesis compared to that in the MDI-treated control group (no. 2). Noteworthily, TG accumulation was not reduced in the YBS1-treated groups (nos. 5, 6, and 7) in the middle and late stages of adipogenesis compared to that in the MDI-treated control group (no. 2). These findings led us to hypothesize that YBS1 is more effective in inhibiting TG accumulation in the early stages of adipocyte differentiation than in the middle or late stages.

### Treatment with YBS1 Supernatant Inhibits Adipocyte Differentiation in 3T3-L1 Pre-Adipocytes via p38 MAPK Signaling

**Fig. 4:**
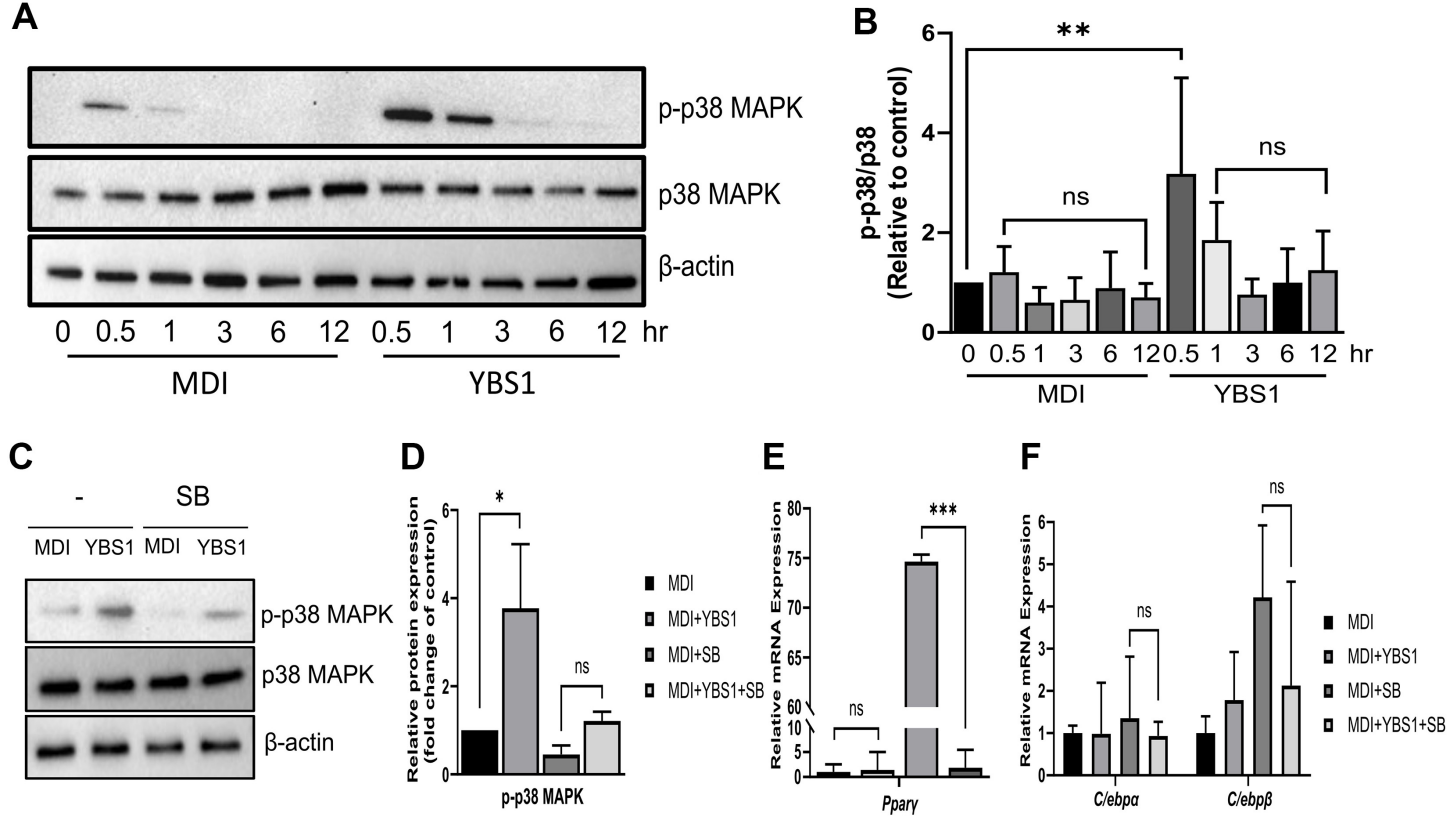
YBS1 inhibits the early differentiation of 3T3-L1 pre-adipocytes through p38 MAPK signaling. (**A and B**) Expression and quantification of p38 MAPK phosphorylation levels between cells treated with and without 5 μl/ml YBS1 for 0.5, 1, 3, 6, and 12 h. (**C and D**) Phosphorylation levels and quantification of p38 MAPK in 3T3-L1 pre-adipocytes treated with YBS1 in combination with or without SB203580 for 1 h. Protein expression level was quantified using the Image J program. (**E and F**) mRNA expression level after treatment with SB203580; ns, *, **, and *** indicate non-significance, *p* < 0.05, *p* < 0.01, and *p* < 0.001, respectively. MDI: 0.5 mM isobutylmethylxanthine, 1 μM dexamethasone, and 10 μg/ml insulin; MRS, de Man, Rogosa, and Sharpe medium (5 μl/ml).

To comprehend the suppressive role of YBS1 during the initial phases of adipogenesis, we examined the internal processes that underlie the inhibition of adipogenesis in 3T3-L1 pre-adipocytes following YBS1 supernatant administration. We studied the impact of p38 MAPK signaling in the presence of YBS1 because it controls adipogenesis in 3T3-L1 pre-adipocytes [27]. The phosphorylation of p38 MAPK was checked in 3T3-L1 pre-adipocytes that were treated with YBS1 for 0.5, 1, 3, 6, and 12 h. When compared to the non-MDI-treated control (Fig. 4A), p38 MAPK phosphorylation was considerably enhanced within 0.5 h following MDI treatment (Fig. 4A and 4B). Upon YBS1 treatment, p38 MAPK phosphorylation was strongly elevated at 0.5 h and this elevation was observed only until 3 h. According to these data, YBS1 treatment during adipogenesis may induce p38 MAPK phosphorylation. To verify p38 MAPK signaling in the context of YBS1 treatment, we used the p38 MAPK inhibitor SB203580 (Fig. 4C and D). As shown in Fig. 4C, upon combined treatment with SB203580 and MDI, signs of p38 MAPK phosphorylation were no longer observed. Nonetheless, in YBS1-treated cells further treated with SB203580, p38 MAPK phosphorylation remained discernible. Furthermore, we examined the expression of important key markers, including PPARγ, C/EBPα, and C/EBPβ, in the presence of the p38 MAPK inhibitor (Fig. 4E and F). Treatment with SB203580 markedly increased PPARγ expression. Conversely, PPARγ expression was nearly completely eliminated with co-treatment of SB203580 with YBS1. We also evaluated the expression of C/EBPα and C/EBPβ with SB203580 treatment. After receiving SB203580 treatment, C/EBPβ expression was restored, whereas YBS1 treatment led to a reduction. However, with SB203580 treatment, a discernible change in C/EBPα expression was absent. These findings imply that the p38 MAPK/PPARγ pathway is the mechanism by which YBS1 treatment prevents 3T3-L1 pre-adipocyte development.

## Discussion

Probiotics provide a diverse range of health benefits, including enhancing nutrient availability, modulating the immune system, and decreasing insulin resistance. However, to determine the precise molecular mechanisms by which probiotics contribute to obesity, further research is warranted [28, 29]. The use of LAB as preventive and therapeutic agents in obesity treatment has been reported [30]. *L. plantarum* KY1032 considerably decreased body weight and total fat mass in treated rats compared to the HFD control group by reducing adipogenic-related key markers in 3T3-L1 cells [31]. Furthermore, *L. plantarum* K10 substantially inhibits in 3T3-L1 differentiation and decreases fat mass in mice [32]. *Lactobacillus gasseri* BNR17 isolated from breast milk prevents rats fed an HFD from becoming overweight [33]. One probiotic strain, *Lactobacillus casei* Shirota, has been shown to ameliorate insulin resistance and glucose intolerance in mice with diet-caused obesity [34]. Collectively, these studies demonstrate that *Lactobacillus* strains are beneficial for enhancing anti-obesity approaches. Thus, we hypothesized that YBS1 may have a positive effect on obesity.

Adipogenesis is the term used to describe the procedure by which pre-adipocytes are transformed into mature adipocytes. This process organizes of distinct steps, including growth arrest, MCE, and terminal differentiation. Adipogenesis is tightly governed by vital transcription factors, including C/EBPβ, C/EBPα, and PPARγ [35, 36]. Under the influence of adipogenic hormonal cocktail treatment that promotes fat cell formation, C/EBPβ expression is stimulated during the early stages of fat cell development (known as MCE). This induction of C/EBPβ can then trigger the activation of other regulators, including C/EBPα and PPARγ, which are important for the early stages of fat cell formation. Thus, the presence of C/EBPα and PPARγ triggers the activation of genes related to fat cell formation and enables the termination of the process known as MCE [37]. Isopanduratin A and other phytochemicals positively affect fat formation through activation of the AMP-activated protein kinase (AMPK)-acetyl-CoA carboxylase (ACC) pathway. Isopanduratin A causes dephosphorylation of p-ERK/ERK signaling, which slows adipogenesis in 3T3-L1 cells by inducing growth arrest [38, 39]. However, the specific processes involved in the regulation of MCE during adipogenesis by LAB remain unclear.

AMPK is involved in multiple biological processes, including adipocyte development. Rhinacanthin decreases adipogenic marker expression, including that of PPARγ, C/EBPα, and aP2, by phosphorylating AMPKα. Moreover, it suppresses adipogenesis by deactivating SREBP-1c, acetyl-CoA carboxylase, Fas cell surface death receptor, and stearoyl-Coenzyme A desaturase 1 [40]. AMPKα activation is widely recognized to inhibit C/EBPα, C/EBPβ, and PPARγ expression during adipogenesis. Prior research has demonstrated that LAB and selective probiotics have beneficial effects on obesity caused by food consumption and an HFD. These effects are achieved by induction the AMPK signaling, which can suppress the expression of two important regulators of fat accumulation, namely PPARγ and SREBP-1c [41, 42]. Additionally, treatment with *B. longum* subsp. *infantis* YB0411 reduced fat accumulation and adipogenic gene expression via the activation of AMPK signaling both in vitro and in vivo [43]. Another study elucidated that *L. plantarum* L-14 extract improved obesity in mice via the Toll-like receptor 2 and AMPK signaling pathways [44]. According to the authors, exopolysaccharide derived from *L. plantarum* L-14 binds to TLR2, reduces chronic inflammation in mice fed an HFD, and has a positive effect on obesity by triggering AMPK signaling. Confirmation of the signal transduction of the early factors that suppress adipocytes is important for future research.

In adipocytes, HSL plays a critical role in TG breakdown, which releases free fatty acids into the circulation [45]. Thus, the promotion of lipolysis via HSL activation may be an additional anti-obesity strategy. Kim *et al*. proposed that by activating AMPK and phosphorylating HSL in 3T3-L1 pre-adipocytes, cell-free extracts of *Lactobacillus fermentum* MG4231 and MG4244 decreased intracellular TG accumulation [46]. We also found that 3T3-L1 cells treated with YBS1 enhanced the expression of lipolysis-related genes ATGL and HSL. Thus, it is reasonable to assume that the suppression of early adipogenesis and the activation of the lipolytic genes ATGL and HSL in late adipogenesis are both related to a decline in intracellular TG and fat formation. However, additional research is required to fully comprehend how YBS1 regulates adipogenesis and lipolysis.

Research on browning, the process by which white adipose tissue transforms into brown adipose tissue, has also been vigorously pursued as a novel approach for treating obesity [47, 48]. Numerous studies have confirmed that LAB promotes browning, which has anti-obesity effects. The administration of *Lactobacillus amylovorus* KU4 improved HFD-induced obesity in mice through the promotion of the expression of brown markers, such as Ucp1 and peroxisome proliferator-activated receptor gamma coactivator-1 alpha (Pgc1α), as well as other thermogenic events, such as an increase in mitochondria in adipocytes [49]. Mice fed an HFD and administered *L. acidophilus* showed body weight reduction [50]. Considering this aspect, we also investigated whether the presence of YBS1 could activate browning through the production of thermogenic Ucp1; however, YBS1 treatment did not affect Ucp1 expression (Fig. S3).

Our study revealed that treatment of YBS1 cell-free supernatant in 3T3-L1 cells can effectively decrease intracellular TG accumulation during pre-adipocyte differentiation. Additionally, this treatment can suppress the expression of the crucial adipogenic gene PPARγ by activating p38 MAPK in the early stages of adipocyte differentiation. Our findings propose that YBS1 positively affects combating obesity; however, further investigation is needed.

## Supplemental Materials

Supplementary data for this paper are available on-line only at http://jmb.or.kr.
