# Supplementary material for: Anti-Obesity Effect of Lactobacillus acidophilus DS0079 (YBS1) by Inhibition of Adipocyte Differentiation through Regulation of p38 MAPK/PPARγ Signaling
Source: J Microbiol Biotechnol. 2024 Mar 29;34(5):1073–81. doi: 10.4014/jmb.2402.02012 (PMC11180917; doi:10.4014/jmb.2402.02012)
Supplement: Supplementary file 1 [file jmb-34-5-1073-supple.pdf]

J. Microbiol. Biotechnol.
https://doi.org/10.4014/jmb.2402.02012

Supplementary Figures and Tables

Anti-Obesity Effect of *Lactobacillus acidophilus* DS0079 (YBS1) by Inhibition of Adipocyte Differentiation through Regulation of p38 MAPK/PPARg Signaling

Youri Lee[1, 2], Navid Iqbal[1, 2], Mi-Hwa Lee[3], Doo-Sang Park[4], and Yong-Sik Kim[1, 2*]

[1] Department of Microbiology, College of Medicine, Soonchunhyang University, Cheonan, Chung-nam 31151, Republic of Korea

[2] Institute of Tissue Regeneration, College of Medicine, Soonchunhyang University, Cheonan, Chung-nam 31151, Republic of Korea

[3] Nakdonggang National Institute of Biological Resources, Sangju 37242, Republic of Korea

[4] Biological Resource Center, Korea Research Institute of Bioscience and Biotechnology, Jeongeup 56212, Republic of Korea

* Corresponding author:

Yong-Sik Kim, Ph.D.

Email: yongsikkim@sch.ac.kr

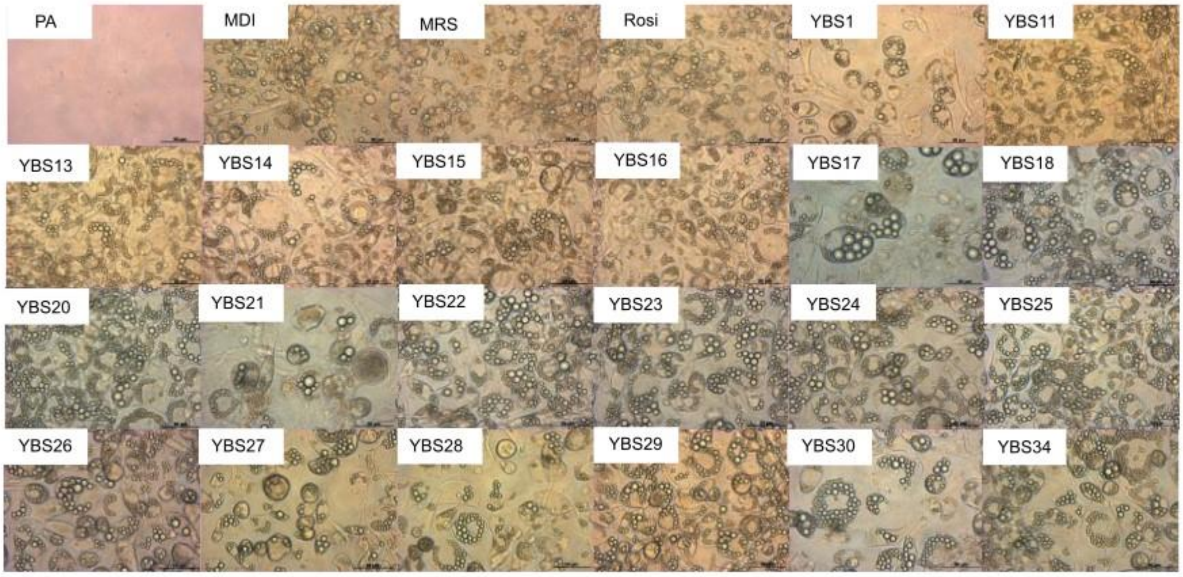

Fig. S1. **Microscopic pictures of *Lactobacillus* culture supernatant-treated 3T3-L1 pre-adipocytes. Magnification is 200×.**

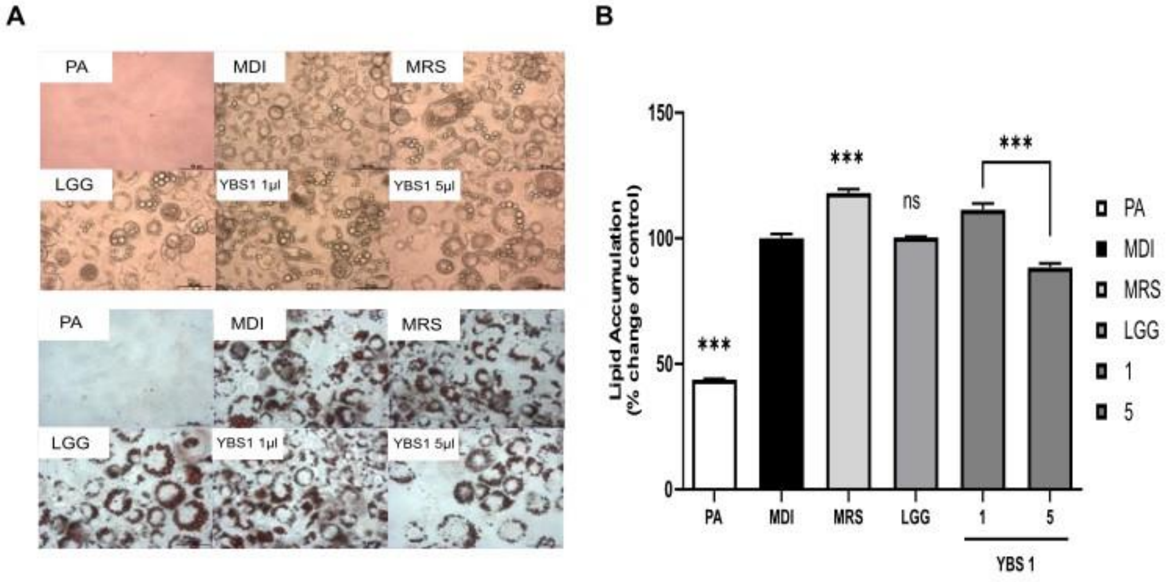

Fig. S2. Triglyceride (TG) accumulation by dose-dependent YBS1 treatment in 3T3-L1 pre-adipocytes. (A) Microscopic pictures and ORO staining pictures with and without YBS1 treatment in 3T3-L1 pre-adipocyte differentiation. **(B)** The measurement of TG accumulation in differentiated 3T3-L1 cells with YBS1 treatment. MDI: 0.5 mM IBMX, 1 µM dexamethasone and 10 µg/mL insulin; 1 µM Rosiglitazone (Rosi); 5 µL/mL MRS; 5 µL/mL YBS1; 5 µL/mL LGG: *Lactobacillus rhamnosus* GG.

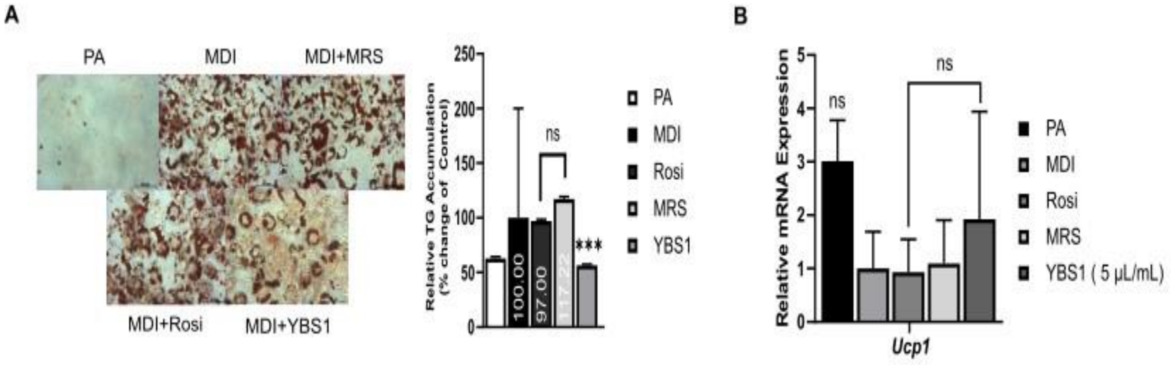

Fig. S3. Triglyceride (TG) accumulation by YBS1 treatment in 3T3-L1 pre-adipocytes. (A) Oil Red O staining microscopic pictures and quantification of TG accumulation. **(B)** The mRNA expression of browning related genes Ucp1. MDI: 0.5 mM IBMX, 1 µM dexamethasone and 10 µg/mL insulin; 1 µM Rosiglitazone (Rosi); 5 µL/mL MRS; 5 µL/mL YBS1.

Supplementary Table S1. List of Lactic acid bacteria in this study.

Serial No.	Strain No.	Strains	Deposit No.
**1**	**DS0079**	***Lactobacillus acidophilus***	**KCTC15515BP**
11	DS0725	*Lactobacillus paracasei*	KCTC15516BP
13	DS1040	*Lactobacillus paracasei*	BP1883436
14	DS0612	*Lactobacillus pentosus*	BP1883339
15	DS0802	*Lactobacillus pentosus*	BP1883371
16	DS0811	*Lactobacillus pentosus*	BP1883379
17	DS0884	*Lactobacillus pentosus*	BP1883399
18	DS1273	*Lactobacillus pentosus*	BP1883703
20	DS0613	*Lactobacillus plantarum*	BP1883340
21	DS0708	*Lactobacillus plantarum*	BP1295036
22	DS0786	*Lactobacillus plantarum*	BP1883390
23	DS0787	*Lactobacillus plantarum*	BP1883391
24	DS0795	*Lactobacillus plantarum*	BP1883364
25	DS0806	*Lactobacillus plantarum*	BP1883374
26	DS0815	*Lactobacillus plantarum*	BP1883382
27	DS0888	*Lactobacillus plantarum*	BP1883383
28	DS1073	*Lactobacillus plantarum*	BP1883494
29	DS1274	*Lactobacillus plantarum*	BP1883526
30	DS1530	*Lactobacillus plantarum*	BP1883589
34	DS0483	*Lactobacillus rhamnosus*	BP1883176

Supplementary Table S2. The primer sequences used for qRT-PCR in this study.

Primer Name	Forward (5'-3')	Reverse (5'-3')
mPparγ	TTTGAAAGAAGCGGTGAACCAC	ACCATTGGGTCAGCTCTTGTG
mC/ebpα	GAGCCGAGATAAAGCCAAACA	CGGTCATTGTCACTGGTCAACT
mC/ebpβ	CAAGCTGAGCGACGAGTACA	CAGCTGCTCCACCTTCTTCT
mC/ebpδ	ACGACGAGAGCGCCATC	TCGCCGTCGCCCCAGTC
maP2	GTGATGCCTTTGTGGGAAACCTG GAAG	TCATAAACTCTTGTGGAAGTCACG CC
mAdiponectin	GATGCAGGTCTTCTTGGT CCTAA	GGCCCTTCAGCTCCT GTC
mAcc	TGACCGTGGGCACAAAGTT	AGGAGGAACCGCATTTATCG
mSrebp1c	GATCAAAGAGGAGCCAGTGC	TAGATGGTGGCTGCTGAGTG
mDgat2	CCGCAAAGGCTTTGTGAA	GGAATAAGTGGGAACCAGATC
mDgat1	GGAATATCCCCGTGCACAA	CATTTGCTGCTGCCATGTC
mFas	GGTTCGGAATGCTATCCAGG	CTGCGGAAACTTCAGGAAAT
mPerillipin	AGAGTTCTGCAGCTGCCTGT	CAGAGGTGCTTGCAATGGGC
mAtgl	CAACGCCACTCACATCTACG	TCACCAAGGTTGAAGGAGGGA
mHsl	ACAGTGCAGGTGGGAATCTC	GCCTAGTGCCTTCTGGTCT
mTbp	GAAGCTGCGGTACAATTCCAG	CCCCTTGTACCCTTCACCAAT
